# Analysis of the beneficial effects of prior soybean cultivation to the field on corn yield and soil nitrogen content

**DOI:** 10.3389/fpls.2024.1413507

**Published:** 2024-07-30

**Authors:** Chao Yan, Yi Yang, Junming Song, Fuxin Shan, Xiaochen Lyu, Shuangshuang Yan, Chang Wang, Qiulai Song, Chunmei Ma

**Affiliations:** ^1^ College of Agriculture, Northeast Agricultural University, Harbin, China; ^2^ Chinese People’s Armed Police Force Non Commissioned Officer School, Hangzhou, China; ^3^ Institute of Crop Cultivation and Tillage, Heilongjiang Academy of Agricultural Sciences, Harbin, Heilongjiang, China

**Keywords:** rotation, straw return, corn yield, nitrogen fractions, ^15^N-labeled

## Abstract

Corn-soybean rotation is a cropping pattern to optimize crop structure and improve resource use efficiency, and nitrogen (N) fertilizer application is an indispensable tool to increase corn yields. However, the effects of N fertilizer application levels on corn yield and soil N storage under corn-soybean rotation have not been systematically studied. The experimental located in the central part of the Songnen Plain, a split-zone experimental design was used with two planting patterns of continuous corn (CC) and corn-soybean rotations (RC) in the main zone and three N application rates of 0, 180, and 360 kg hm^-2^ of urea in the secondary zone. The research has shown that RC treatments can enhance plant growth and increase corn yield by 4.76% to 79.92% compared to CC treatments. The amount of N fertilizer applied has a negative correlation with yield increase range, and N application above 180 kg hm^-2^ has a significantly lower effect on corn yield increase. Therefore, a reduction in N fertilizer application may be appropriate. RC increased soil N storage by improving soil N-transforming enzyme activity, improving soil N content and the proportion of soil organic N fractions. Additionally, it can improve plant N use efficiency by 1.4%-5.6%. Soybeans grown in corn-soybean rotations systems have the potential to replace more than 180 kg hm^-2^ of urea application. Corn-soybean rotation with low N inputs is an efficient and sustainable agricultural strategy.

## 1 Introduction

A two-year crop rotation of corn and soybean is one of the most widely used crop rotation systems in single-crop areas, and this system can increase crop yield, improve soil quality, and reduce environmental pollution. In particular, the inclusion of soybean in a crop rotation enhances biological nitrogen (N) fixation (BNF) in root nodules, provides an effective source of fertilizer for the succeeding crop, and establishes a high-quality elemental cycling system, thus improving soil fertility and alleviating soil fertility degradation. In large-scale agricultural production regions in the United States (US), most farmland is managed by crop rotation (Wallander, 2013). According to the US Department of Agriculture’s Agricultural Resource Management Survey (ARMS), approximately 18% of cropland is continuously planted to corn.

By enhancing biodiversity, the corn-soybean rotation system maintains the carbon and N balance of farmland soils and promotes the synergistic development of ecosystems (Shah et al., 2021). Some scholars consider corn to be a crop that is tolerant to continuous cropping, however, maintaining high yields of corn under continuous cropping requires the continuous input of large amounts of chemical N fertilizer (Gentry et al., 2013). Soybean plants can fix N from the air into the soil for their own use or for use by succeeding crops. Some studies have shown that 60% of the N taken up by soybean plants comes from BNF in root nodules (Ohwaki and Sugahara, 1997), and the remaining N fixed in the soil by rhizobia and N from soybean plant residues can be used by succeeding crops and for the improvement of soil fertility (Hoagland et al., 2008). Beans can fix approximately 39-182 kg N ha^−1^ (Peoples et al., 2009), and the effect of the corn-soybean rotation system is more pronounced in low fertilizer input systems (Mahmoud et al., 2022). In the corn-soybean rotation system, the decomposition period of corn straw overlaps with the growth period of soybean. Due to the intense deposition of N during straw decomposition, plants in this system fix more N than do those in continuous cropping systems (per plant) (Xu et al., 2020), thus significantly improving the soil nutrient use efficiency (Miyazawa et al., 2010; Tang et al., 2014).

Northeast China is an important corn production base. In this region, corn has been grown continuously cultivated for a long time, the fertilizer input is high, and the N use efficiency (NUE) is significantly lower than the global average. Corn-soybean crop rotation is considered a diversified farming system (DFS) and an effective means for adjusting agricultural cropping structure and improve resource use efficiency. Based on the corn-soybean rotation system and long-term experiments of continuous corn cropping, this study investigated the effects of different N fertilizer input amounts on corn yield and soil N stocks, clarified the effect of using soybean as the preceding crop on the yield increase of subsequent corn crops and soil N stocks, and provides a reference for corn production in Northeast China.

## 2 Materials and methods

### 2.1 Overview of the test sites

**Figure 1:**
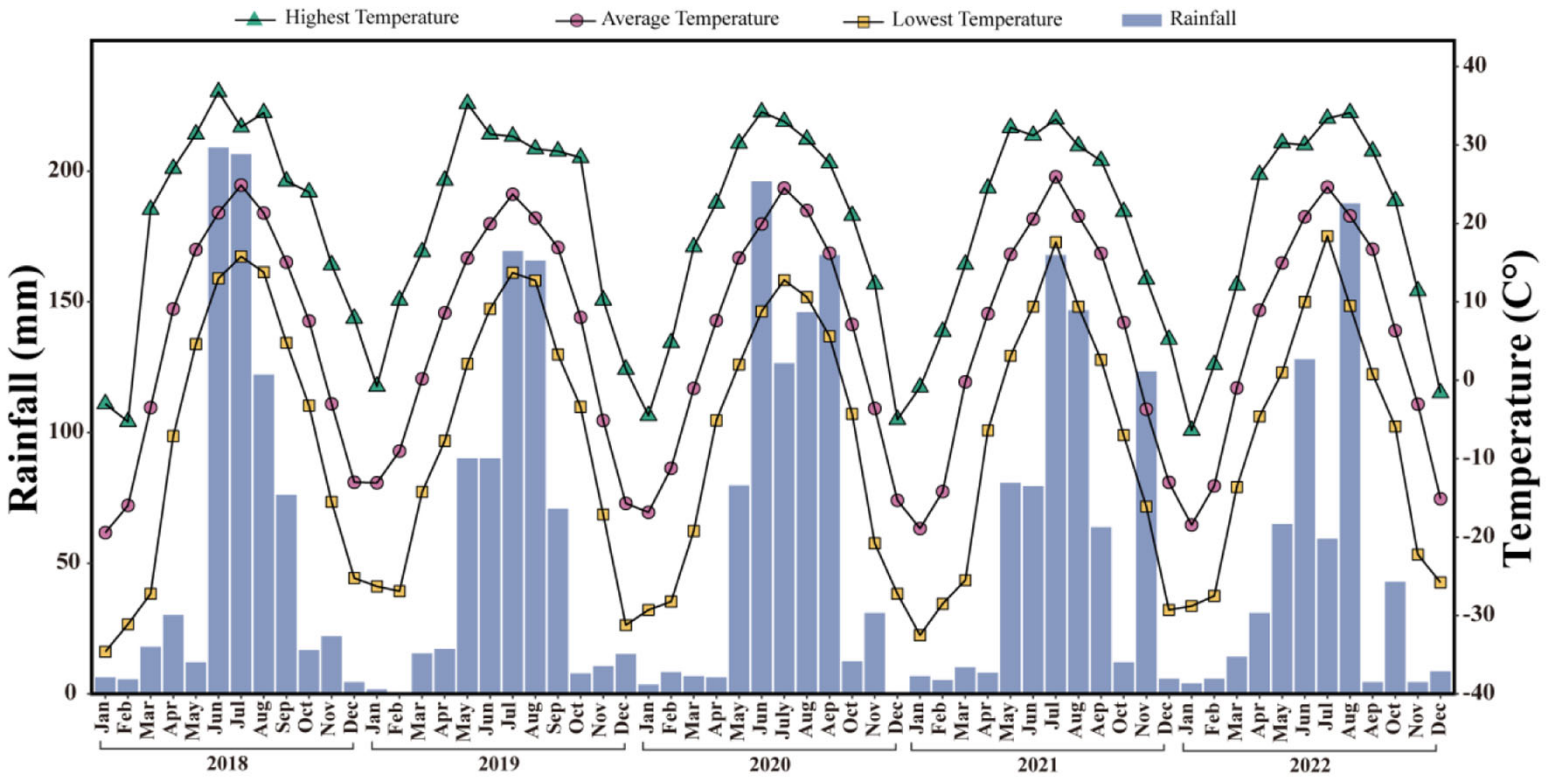
Temperature and precipitation during the experimental period.

The field experiment was conducted at the Xiangyang Experimental Practice Base (123°22′-126°50′E; 45°34′-45°46′N) of Northeast Agricultural University in Harbin city, Heilongjiang Province, China. The monthly average temperature and rainfall during the experimental period are shown in **Figure 1**. Located in the central part of the Songnen Plain, the region has a cool temperate continental climate with simultaneous hot and rainy seasons occurring simultaneously. During the experiment, the mean annual average temperature was 5.4°C, the frost-free period was approximately 141 days, the average annual rainfall was 687 mm, and the annual average number of days with daily average temperature ≤0°C was 129. The active cumulative temperature ≥10°C was above 2760°C. The chemical properties of the 0-30 cm soil layer in the experimental plot were as follows: soil organic matter (SOM), 34.29 g kg^−1^; total N (TN), 1.92 g kg^−1^; total phosphorus (TP), 0.51 g kg^−1^; total potassium (TK), 21.60 g kg^−1^; available N, 100.14 mg kg^−1^; available phosphorus, 33.82 mg kg^−1^; and available potassium, 246.49 mg kg^−1^; pH = 6.14.

### 2.2 Experimental design and field management

**Figure 2:**
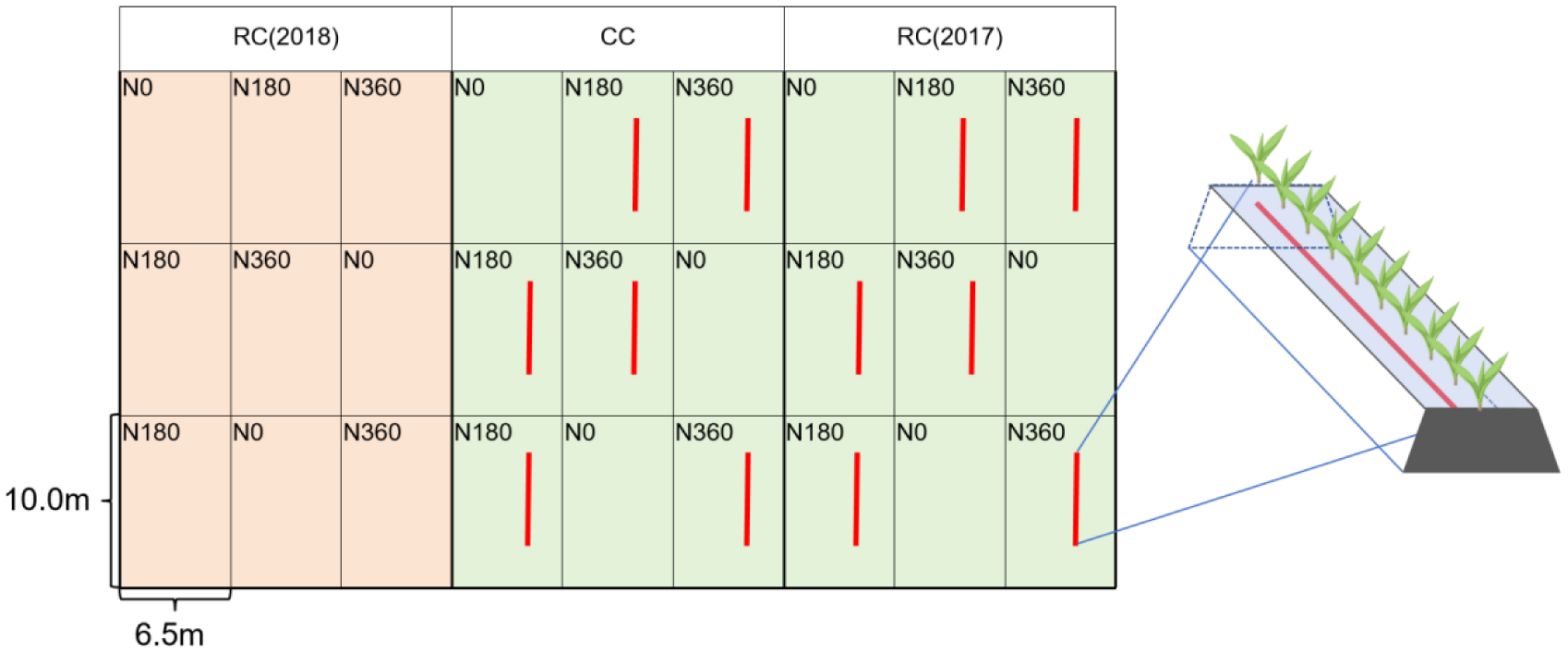
Diagram of the field arrangement used in the experiment. The red line represents the application of ^15^N-labeled urea in 2022.

The long-term experiment was arranged in the autumn of 2017, and the associated study started in 2018. The split-plot experimental design was adopted, the main plots were for continuous cropping (CC) and corn-soybean rotation cropping (RC), and the secondary plots had three N fertilization levels (N0, N180, and N360). Each plot had 10 ridges, the ridge length was 10.0 m, the ridge spacing was 0.65 m, and the area was 65.0 m^2^. Each plot had 3 replicates for each experiment. To exclude interannual environmental differences, two corn-soybean rotation experimental areas were established, i.e., RC (2017): soybean (Kenfeng 16) was planted in 2017, and corn (Guoyu 49) and soybean were planted every other year beginning in 2018; RC (2018): corn stubble was left in the field in 2017, and soybean and corn were planted every other year beginning in 2018. The field arrangement is shown in **Figure 2**. The specific fertilizer rates for the corn and soybean fields are shown in **Table 1**.

**Table 1:** N application rates (kg hm^-2^).

Crop	Corn			Soybean		
	Urea(N:46%)	concentrated superphosphate (P_2_O_5_:44%)	potassium sulphate (K_2_O:50%)	Urea(N:46%)	concentrated superphosphate (P_2_O_5_:44%)	potassium sulphate (K_2_O:50%)
N0	0	150	75	0	150	75
N180	180	150	75	30	150	75
N360	360	150	75	60	150	75

The seeds were sown around 5 May every year. Sowing was done manually. Fertilizer was applied in a single pass after ridges were ploughed open and then the ridges were closed to bury the fertilizer There were 60,000 corn plants/hm^2^ and 300,000 soybean plants/hm^2^. The harvest occurred around October 5. The management measures used were consistent with those used in local field production. In 2022, two main plots, CC and RC (2017), were selected for the ^15^N labeling test, which was done by arranging a ^15^N labeling trial in a randomly selected ridge with a ridge length of 2.5 m within the plots of the two fertilizer treatments (N180 and N360) and applying ^15^N labelled urea (abundance: 3.603%). **Figure 2** shows the detailed field arrangement.

### 2.3 Sample collection and analysis

#### 2.3.1 Soil sample collection and analysis

##### 2.3.1.1 Soil sampling

After the 2022 harvest, soil samples were collected from two soil layers, 0-15 cm (upper layer) and 15-30 cm (lower layer), within a ridge using a 3 cm diameter soil auger. For each plot, soil samples were collected from 5 sampling points, mixed into one sample, and residues such as plant roots were manually removed. Each treatment had 3 replicates; the soil samples were thoroughly mixed and then divided into two parts: one was air dried and used for the determination of soil organic N fractions and TN; and the other was stored in a -20°C freezer for the determination of soil inorganic N. In addition, undisturbed soil from two soil layers, 0-15 cm (upper layer) and 15-30 cm (lower layer), was collected with a 100 cm^3^-ring-knife soil sampler for analysis of soil bulk density.

##### 2.3.1.2 Determination of TN in soil

One gram of air-dried soil was passed through a 100-mesh sieve, concentrated sulfuric acid and a mixed catalyst were added for digestion at 360-410°C, and the TN content was measured by the Kjeldahl N analyzer.

##### 2.3.1.3 Determination of soil organic N fractions

According to the methods described by Bremner (Bao, 2000), an air-dried soil sample containing approximately 10.00 mg of N was weighed, mixed with 2 drops of n-octanol and 20 mL of 6 mol/L HCl, and subsequently hydrolyzed at 120 ± 3°C for 12 h on a hot plate. After hydrolysis, filtration was performed, the filtrate was neutralized until the pH reached 6.5 ± 0.1, and the solution was diluted to 100 mL. MgO was added to the acidolysis solution and distillation was carried out to obtain the acid hydrolysed ammonia N (AN) content. The acidolysis solution was added to borate-phosphate buffer for distillation, and by subtracting the AN content from the obtained result, the acid amino sugar N (ASN) content was obtained. Concentrated sulfuric acid and catalyst were added to the acidolysis solution for digestion at 200°C in the digestion furnace, followed by distillation, and the total soil acid hydrolyzed N (THAN) content was obtained. The alkaline hydrolysis solution was treated with NaOH and then placed in a water bath at 100°C after adding citric acid and ninhydrin. Finally, the borate-phosphate buffer was added for distillation to obtain acid hydrolyzed amino acid N (AAN); the acid hydrolyzed unknown N (HUN) content was obtained by subtracting AN, ASN and AAN from THAN; and the non-acid hydrolyzed N content was obtained by subtracting THAN from soil TN.

##### 2.3.1.4 Determination of soil mineral N

The KCl extraction-distillation method was used. First, 10.00 g of fresh soil was weighed and extracted with 50 mL of 2 mol/L^-1^ KCl; 0.72 g of MgO was added to part of the extract and the soil NH_4_^+^-N concentration was determined by distillation using a Kjeldahl N analyser; the mixture of 0.72 g of MgO, 0.6 g of FeSO4 and zinc powder was added to another part of the extract, and the soil mineral N concentration was measured using a Kjeldahl N analyzer. The soil NO_3_^-^-N concentration was calculated by subtracting the NH_4_^+^-N concentration from the mineral N concentration.

##### 2.3.1.5 Determination of soil microbial biomass N (MBN)

The chloroform fumigation method (Vance et al., 1987) was used. After the extraction of soil samples fumigated with chloroform for 24 h, the organic N content was determined by a Kjeldahl N analyzer. Soil MBN content was obtained by dividing the difference in organic N content between fumigated and unfumigated soil by kEN (0.57).

Soil enzyme activity was determined using a soil enzyme kit from Solarbio Biotech Co., Ltd., Beijing, China (Ref.). The kit included tests for urease (product No. BC0125), nitrate reductase (product no. BC3105), nitrite reductase (product no. BC2995), leucine aminopeptidase (product no. BC4025), and N-acetyl-β-D-glucosidase (product no. BC4005).

#### 2.3.2 Collection and analysis of plant samples

On 19 June (seedling stage), 8 July (jointing stage), 1 August (tasseling stage), 17 August (silking stage), 27 August(grain-filling stage), 13 September (milk stage) and 3 October (maturity stage) of 2022, plant samples were collected, fixed at 105°C for 30 min, dried at 80°C, weighed for dry matter weight, and subsequently crushed and digested with concentrated sulfuric acid. The N content of the plant was determined by a Kjeldahl N analyzer. The titrated sample solution was concentrated and reacted with lithium hypobromite under refrigerated vacuum conditions to produce the N_2_, and the ^15^N abundance was determined by isotope ratio mass spectrometer (Thermo-Fisher, USA) using dual channel (DI) measurements.

#### 2.3.3 Yield measurement

During the corn maturity stage, an area of 5 m long and 1.3 m wide (2 ridges wide) was selected as the sampling area in each plot. All ears in the sampling area were harvested. After air-drying, the water coefficient was measured, and the yield was converted with the water content being 14%.

### 2.4 Relevant calculations

The calculation method of the TN stocks and increase for a corresponding soil layer refers to Yan et al. (2023). The TN stocks was calculated as:


(1) $$N_{stock\;}=\;TN_{stock}\times BDi\times Hi$$

where N_Stock_ indicates the TN_stock_ (kg hm^−2^) and BDi and Hi indicate the soil bulk density (g cm^−3^) and the thickness (m) of the corresponding soil layer, respectively. The increase in soil N stock was calculated as:


(2) $$\Delta {\text{N}}_{\text{stock}}=({\text{N}}_{\text{stock}}\left(\text{RC}\right)-{\text{N}}_{\text{stock}}\left(\text{CC}\right)/\text{t}$$

ΔN_Stock_ is the increase in soil N stock, N_Stock_(RC) is the soil N stock under the RC treatment, N_Stock_(CC) is the soil N stock under the CC treatment, and t is the year. The calculation method of nitrogen use efficiency was referred to Rocha et al. (2019). The proportion of N in the sample from fertilizer was calculated as:


(3) $$\text{Ndff }=\;\frac{{\text{f}}_{\text{t}}-{\text{f}}_{0}}{{\text{f}}_{\text{N}}-{\text{f}}_{0}}$$

Ndff: proportion of N in the sample from fertilizer; f_t_: ^15^N abundance of the treated sample; f_0_: natural abundance of ^15^N, 0.365%; f_N_: ^15^N abundance in the fertilizer, 3.603%. The N accumulation was calculated as:


(4) W = m× Q

where W is the N accumulation in the sample in mg; m is the sample dry weight in g; and ρ is the N content in the sample in mg g^-1^. The plant N accumulation from the fertilizer was calculated as:


(5) $${\text{NC}}_{\text{f}}\;=\;\sum_{i}^{n}{\text{W}}_{\text{i}}\times \text{Ndff}$$

where NC_f_ is the plant N accumulation from the fertilizer in mg plant^-1^, Wi is the N accumulation in different organs of the plant in mg, and Ndffi is the proportion of N from the fertilizer in the corresponding organ. The N use efficiency was calculated as:


(6) $$\text{NUE}\left(\%\right)\;=\;\frac{{\text{NC}}_{\text{f}}}{{\text{N}}_{\text{f}}}\times 100$$

NUE: N use efficiency; N_f_: N fertilizer rate, unit: mg plant^-1^. The straw-grain ratio was calculated as:


(7) $$\text{Straw}-\text{grain ratio}=\frac{\left(\text{m}1+\text{m}2\right)}{\text{M}}$$

where, m1 is the dry weight of the plant stalk in g plant^-1^, m2 is the dry weight of the plant leaves in g plant^-1^, and M is the dry weight of the plant grains in g plant^-1^.

### 2.5 Data analysis

SPSS 18.0 software (IBM Software, Chicago, IL, USA) was used for data management and multivariate analysis. The means were compared by one-way analysis of variance (ANOVA), P ≤ 0.05. The Pearson correlation coefficient was calculated to determine the correlations between indicators. Origin 2022 software (OriginLab, Northampton, MA, USA) was used to plot figures. Nomenclature

## 3 Results

### 3.1 Corn yield

Based on the analysis of five years of yield data, both N application and preceding soybean cultivation significantly increased corn yield (**Table 2**), and corn yield varied significantly among years, ranging from 4,468.26 to 11,040.52 kg hm^-2^. Under the CC treatment, the yield increase under the N180 fertilization treatment was 32.07%-87.85%, with an average yield increase of 55.72% compared with that under the N0 fertilization treatment. Similarly, the yield increase under the N360 fertilizer treatment compared to the N0 fertilizer treatment was 38.25%-93.19%, with an average yield increase of 65.50%. Under the same N application rate, compared with the CC treatment, the RC treatment significantly increased the corn yield. Under the N0 fertilization, the yield increase with preceding soybean plants was 31.13%-79.92%, with an average yield increase of 43.27%. The yield increase of the crop rotation system under the N180 fertilization treatment was in the range of 4.76%-39.03%, with an average yield increase of 14.12%. The rotation yield increase under the N360 fertilizer treatment ranged from 7.06% to 36.67%, with an average yield increase of 16.85%. Analysis of yield components showed that the yield increase resulting from crop rotation was mainly due to a significant increase in the diameter of corn ears, while the effect of fertilization on yield components was manifested as a significant increase in ear length, ear diameter and 100-kernel weight (**Supplementary Figure 1**). A comprehensive analysis of the yield-enhancing effect of crop rotation and N fertilization showed that the corn yield in the treatment with preceding soybean plants and no N application was equivalent to that of continuously cropped corn at the N180 level.

**Table 2:** Corn yield (kg hm^-2^) and yield increase (%).

Year	Treatments	N0	N180	N360	Nitrogen fertilizer yield increase rate	Nitrogen fertilizer yield increase rate
2018	CC	6428.79 ± 215.93cB	8490.77 ± 48.86bB	9679.25 ± 69.96aB	32.07	50.56
	RC	8489.85 ± 75.24cA	9424.90 ± 104.57bA	11040.52 ± 171.41aA	11.01	30.04
	RC yield increase rate	32.06	11.00	14.06		
2019	CC	5242.60 ± 61.84bB	9034.97 ± 110.91aA	9171.52 ± 89.20aB	72.34	74.94
	RC	7242.50 ± 82.61cA	9465.37 ± 125.25bA	10080.72 ± 15.56aA	30.69	39.19
	RC yield increase rate	38.15	4.76	9.91		
2020	CC	4468.26 ± 74.26bB	8393.78 ± 83.16bB	8632.24 ± 99.08aB	87.85	93.19
	RC	6035.89 ± 118.33bA	8935.46 ± 78.40aA	9241.92 ± 58.79aA	48.04	53.12
	RC yield increase rate	35.08	6.45	7.06		
2021	CC	6441.54 ± 127.14bB	8653.85 ± 37.09aB	8905.64 ± 38.98aB	34.34	38.25
	RC	8446.92 ± 100.17cA	9461.54 ± 33.31bA	10380.00 ± 120.00aA	12.01	22.88
	RC yield increase rate	31.13	9.33	16.56		
2022	CC	4306.56 ± 105.21bB	6545.58 ± 279.85aB	7343.80 ± 196.04aB	51.99	70.53
	RC	7748.26 ± 216.22cA	9100.36 ± 280.50bA	10036.96 ± 148.63aA	17.45	29.54
	RC yield increase rate	79.92	39.03	36.67		
Year (Y)		**				
Treatment (T)		**				
nitrogen fertilizer (N)		**				
Y×T		**				
Y×N		**				
T×N		**				
Y×T×N		ns				

Lowercase letters represent significant differences (p<0.05) between different N application rates for the same treatment in the same year; uppercase letters represent significant differences (p<0.05) in corn yield between RC and CC under the same N application rate in the same year; ns, not significant; ** indicate significant differences at the 0.01 levels, respectively.

**Figure 3:**
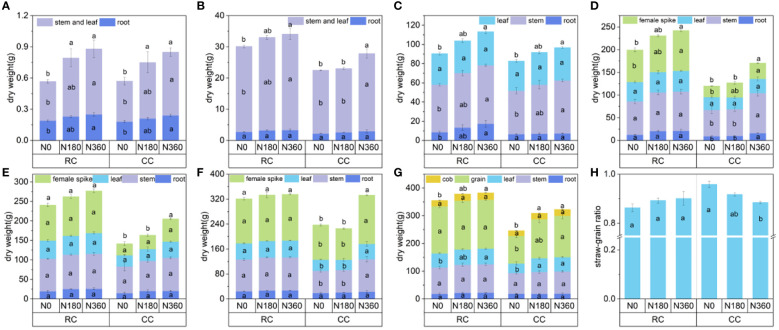
Changes in the dry matter weight and grass-grain ratio of maize plant. **(A–G)** are the dry matter weight of June 19, 2022 (seedling stage), July 8, 2022 (jointing stage), August 1, 2022 (tasseling stage), August 17, 2022 (silking stage), August 27, 2022 (grain-filling stage), September 13, 2022 (milk stage), October 3, 2022 (maturity stage), **(H)** is the grass-to-grain ratio of mature plants. The lowercase letters are the significant differences between different fertilization gradients in the same stubble, p < 0.05.

Samples of corn were taken in 2022 during the seven main growth periods. Analysis of the dry matter weight showed that the treatment with preceding soybean cultivation and N fertilization significantly increased the dry matter weight of the corn plants to different degrees (**Figure 3**), and the dry matter weight increased gradually with increasing fertilization application rate. On 19 June (corn seedling stage), the dry weights of the shoots and underground parts of corn plants under the N360 and N180 treatments were significantly greater than those under the N0 treatment, and there was no significant difference between the RC and CC treatments under the same N level (**Figure 3A**). After 8 July, the dry weight in the RC treatment was significantly greater than that in the CC treatment at the same N fertilization level. During this period, the preceding cultivation of soybean plants promoted the growth and dry matter accumulation of corn plants, and the dry weight increased with increasing fertilizer application rate (**Figures 3B–G**). As shown in **Figures 3D, E**, the dry weight of the ears of corn in the RC treatment was between 70.85 g and 107.97 g, accounting for 34.65%-38.96% of the dry weight of the whole plant; the dry weight of the ears in the CC treatment was between 24.21 g and 58.51 g, accounting for 20.18%-28.50% of the dry weight of the whole plant. On 13 September (the milk stage), there was no significant difference between the different N levels in the RC treatment, and the dry weight of plants at the N360 level of the CC treatment was significantly higher than that of plants at the N180 and N0 levels, with the difference in dry weight ranging from 95.43g to 107.07g (**Figure 3F**). On 3 October (the maturity stage), the dry matter weight in the RC treatment was 59.6-125.8 g/plant greater than that in the CC treatment at the same fertilization level, with an average increase of 85.0 g/plant, and notably, the seed dry weight at the N0 level under the RC treatment was comparable to that at the N360 level under CC treatment. As is seen with Equations (7) In the RC treatment, differences in the straw-grain ratio among different N fertilizer levels were not significant; in the CC treatment, the straw-grain ratio gradually decreased with increasing N application rate, and that at the N0 level was significantly higher than that at the N360 level, suggesting that more dry matter accumulated in the stalks when the N supply was insufficient (**Figure 3H**).

### 3.2 Plant N content and NUE

**Figure 4:**
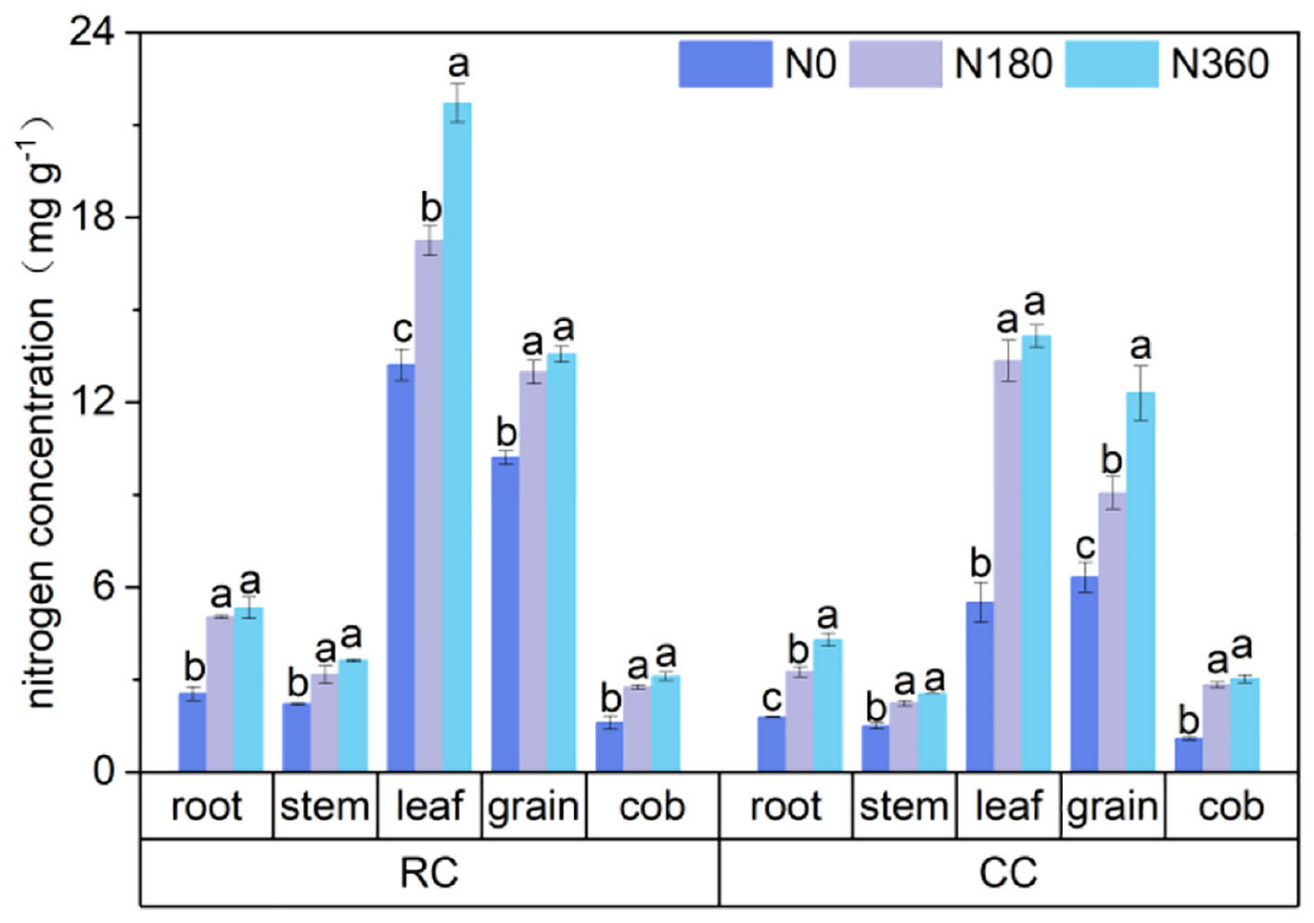
N contents in plants. The lowercase letters are the significant differences between different fertilization gradients in the same stubble, p < 0.05.

The leaves and grains of the corn plants had the highest N contents, and the preceding cultivation of soybean and N fertilization significantly increased the N contents in different parts of the corn plants (**Figure 4**), but did not change the proportion of N in each organ. The N content of each organ of the corn plant increased with increasing N application rate, and at the same N level, the N content in the same plant part was significantly higher in RC treatment than in CC treatment. Nitrogen fertilization and crop rotation had greater effects on leaf and grain N contents. In the CC treatment, the N content in kernels was higher than that in leaves at the N0 level, and the N content in the parts of the corn plant in the other treatments was in the order of leaves, kernels, rootstalks, and ears, suggesting that there was a transfer of N from the leaves to the kernels in the CC treatment. In the RC treatment, the differences in the N content of the corn leaves between the three N fertilization levels were significant, while the N contents at the other locations were not significantly different between the N360 and N180 treatments and were significantly greater than those at the N0 level. In the CC treatment, the differences in the N contents in the corn roots, stalks and grains between the three N fertilization levels were significant, while the N contents in the leaves and ears were not significantly different between the N360 and N180 treatments and were significantly higher than those in the N0 treatment.

As corn grew, the abundance of ^15^N in the plants gradually decreased, indicating that corn absorbed more N in the early growth phase (**Table 3**). Between 19 June and 1 August, the difference in ^15^N abundance in the same part of the corn plants under the different N levels in the same treatment was not significant. However, on 19 June, the ^15^N abundance in the shoots of the RC treatment was significantly lower than that of the CC treatment, indicating that the uptake of soil N by the corn plants occurred earlier in the RC treatment than in the CC treatment. On 27August, the ^15^N abundance in the corn roots at the N360 level was significantly higher than that at the N180 level, and under the same N application level, the ^15^N abundance in the corn roots and stems in the CC treatment was significantly higher than that in the RC treatment. On 13 September, the pattern of ^15^N abundance in corn roots, stems and ears was consistent among treatments, showing that the ^15^N abundance at the N360 level was significantly higher than that at the N180 level for the same treatments, and the ^15^N abundance in the CC treatment was significantly higher than that in the RC treatment at the same N application level. At the mature stage and at the N180 and N360 levels, the ^15^N abundance in each part of the corn in the RC treatment was significantly lower than that in the CC treatment.

**Table 3:** ^15^N abundance.

Year	Treatments	N180		N360		N	T	N*T
		RC	CC	RC	CC			
June 19th (seedling stage)	root	2.04 ± 0.04aA	1.87 ± 0.06aA	2.12 ± 0.24aA	2.04 ± 0.15aA	ns	ns	ns
	above ground	1.24 ± 0.30aB	2.93 ± 0.3aA	1.53 ± 0.17aB	3.15 ± 0.07aA	ns	**	ns
June 19th (seedling stage)	root	1.19 ± 0.12aA	0.90 ± 0.12aA	1.46 ± 0.05aA	1.29 ± 0.15aA	*	ns	ns
	above ground	1.10 ± 0.16aA	0.81 ± 0.06bA	1.46 ± 0.15aA	1.28 ± 0.13aA	*	ns	ns
August 1st (heading stage)	root	0.81 ± 0.11aA	1.09 ± 0.07aA	0.98 ± 0.10aA	1.24 ± 0.04aA	ns	**	ns
	stalk	0.74 ± 0.08aA	0.97 ± 0.09aA	0.85 ± 0.10aA	1.05 ± 0.04aA	ns	*	ns
	leaf	0.79 ± 0.11aA	1.04 ± 0.08aA	0.96 ± 0.11aA	1.15 ± 0.01aA	ns	*	ns
August 17th (silking period)	root	0.83 ± 0.05aA	0.97 ± 0.06bA	0.87 ± 0.02aB	1.21 ± 0.05aA	*	**	ns
	stalk	0.75 ± 0.03aA	0.90 ± 0.05bA	0.99 ± 0.13aA	1.18 ± 0.01aA	**	*	ns
	leaf	0.85 ± 0.07aA	1.00 ± 0.07aA	1.00 ± 0.1aA	1.19 ± 0.10aA	ns	ns	ns
	female spike	0.75 ± 0.04aA	0.85 ± 0.04aA	0.90 ± 0.12aA	1.10 ± 0.07aA	ns	ns	ns
August 27th (filling stage)	root	0.69 ± 0.07bB	0.94 ± 0.07bA	0.90 ± 0.08aB	1.08 ± 0.03aA	**	**	ns
	stalk	0.64 ± 0.06aB	0.90 ± 0.02aA	0.79 ± 0.06aB	0.94 ± 0.01aA	*	**	ns
	leaf	0.70 ± 0.09aA	0.89 ± 0.08bA	0.98 ± 0.08aA	1.11 ± 0.04aA	*	ns	ns
	female spike	0.61 ± 0.04aB	0.82 ± 0.04aA	0.86 ± 0.07aA	0.88 ± 0.03aA	*	ns	ns
September 13th (milk ripening stage)	root	0.64 ± 0.05bB	0.98 ± 0.03aA	0.92 ± 0.01aA	1.03 ± 0.04aA	**	**	*
	stalk	0.58 ± 0.04bB	0.86 ± 0.05aA	0.75 ± 0.02aB	0.95 ± 0.03aA	*	**	ns
	leaf	0.67 ± 0.06aA	0.91 ± 0.05aA	0.89 ± 0.07aA	1.05 ± 0.07aA	*	*	ns
	female spike	0.50 ± 0.01bB	0.74 ± 0.00aA	0.73 ± 0.00aB	0.85 ± 0.01aA	**	**	**
October 3rd (full-ripe stage)	root	0.63 ± 0.03bB	0.93 ± 0.10aA	0.87 ± 0.05aB	1.10 ± 0.02aA	*	**	ns
	stalk	0.62 ± 0.04bB	0.76 ± 0.07aA	0.81 ± 0.02aB	0.89 ± 0.10aA	ns	*	ns
	leaf	0.63 ± 0.02bB	0.74 ± 0.08bA	0.88 ± 0.02aB	1.20 ± 0.07aA	**	*	ns
	grain	0.60 ± 0.03bB	0.83 ± 0.08aA	0.76 ± 0.02aB	0.91 ± 0.05aA	ns	*	ns
	spikestalk	0.58 ± 0.07bB	0.84 ± 0.08aA	0.75 ± 0.05aB	0.87 ± 0.03aA	ns	*	ns

Lowercase letters represent significant differences (p<0.05) between different N application rates for the same treatment in the same year; uppercase letters represent significant differences (p<0.05) in corn yield between RC and CC under the same N application rate in the same year; ns means not significant; * and ** indicate significant differences at the 0.05 and 0.01 levels, respectively.

**Figure 5:**
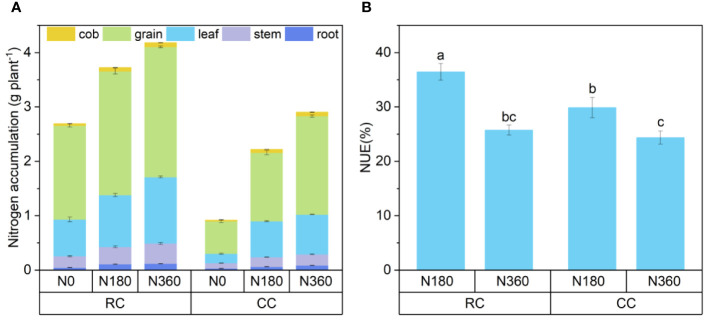
Plant N accumulation and NUE. **(A)** is the nitrogen accumulation in different parts of the maize plant at the mature stage, **(B)** is the NUE of the plant under the nitrogen application treatment, and the lowercase letters are the significant differences in the nitrogen utilization rate under the four treatments, p < 0.05.

The nitrogen accumulation in plant samples was calculated by Equation (4). The N accumulation and N content in the corn plants were similar between the treatments. Under the same N fertilization level, N accumulation in the RC treatment was significantly greater than that in the CC treatment, and N accumulation in each part gradually increased with increasing N fertilization rate (**Figure 5A**). The N accumulation in plants followed the order of grain > leave > stalk > root > ear, and the grain N accumulation accounted for 56.7-65.0% of the total accumulation. Under the CC treatment, grain N accumulation at the N0 level accounted for the highest proportion (65.0%), indicating that when N is insufficient, the N accumulated in the plant is preferentially supplied to the grains. As is seen with Equations (3), (5) and (6). The NUE calculated by ^15^N labelling technology (**Figure 5B**) showed that the NUE ranged from 24.36%-36.45%. Under the N180 and N360 levels, the NUE in the RC treatment was 5.6% and 1.4% greater than that in the CC treatment, respectively. Crop rotation can improve soil NUE, especially in cropping systems with low N inputs.

### 3.3 Changes in soil N

Crop rotation and N fertilization significantly increased the soil N components and the mineral N and TN contents, and these increased with increasing the N fertilizer application rates (**Table 4**). The soil N content in the 0-15 cm soil layer was significantly greater than that in the 15-30 cm soil layer. The soil TN and non-acid hydrolyzed N contents in the RC treatment increased by 0.25-0.44 g kg^-1^ and 0.14-0.31 g kg^-1^, respectively, in the 0-15 cm soil layer, and increased by 0.28-0.39 g kg^-1^ and 0.12-0.24 g kg^-1^, respectively, in the 15 cm-30 cm layer, compared to those in the CC treatment. The amino acids N, ASN, AN, and HUN in the RC treatment increased by -11.49-91.96 mg kg^-1^, 5.02-39.42 mg kg^-1^, 67.4-127.24 mg kg^-1^, and -53.66-13.85 mg kg^-1^, respectively, in the 0-15 cm soil layer, and increased by 5.5-106.76 mg kg^-1^, -34.81-8.18 mg kg^-1^, 60.22-74.8 mg kg^-1^, 20.5-59.18 mg kg^-1^, and 116.11-233.63 mg kg^-1^, respectively, in the 15 cm-30 cm layer, compared with those in the CC treatment. The proportions of soil organic N components in different soil layers changed little. Overall, the stability of the organic N components in the 0-15 cm soil layer was greater than that in the other layers (**Supplementary Figure 2**). The MBN in the 0-15 cm soil layer was significantly greater than that in the 15-30 cm layer, the soil MBN in the RC treatment was greater than that in the CC treatment, and the patterns in the upper and lower soil layers were consistent, indicating that there was no significant pattern in the effect of N fertilization on MBN.

**Table 4:** Soil N components.

Soil depth	Treatment	Nitrogen level	TN	NHN	Acidolysis total nitrogen				MBN	Soil mineral nitrogen	
					AAN	ASN	AN	UN		NH_4_^+^-N	NO_3_^−^-N
			g kg^-1^		g kg^-1^						
0-15cm	RC	N0	2.62 ± 0.03cA	1.48 ± 0.03bA	266.83 ± 2.66cB	168.35 ± 2.18cA	429.04 ± 4.49cA	235.96 ± 1.28cA	19.86 ± 8.21aA	5.20 ± 0.39cA	22.87 ± 0.03cA
		N180	2.78 ± 0.04bA	1.55 ± 0.04bA	352.30 ± 2.27bA	189.65 ± 2.45bA	450.51 ± 0.6bA	270.74 ± 4.70bA	32.63 ± 10.54aA	9.58 ± 0.17aA	24.27 ± 0.02bA
		N360	3.26 ± 0.05aA	1.79 ± 0.05aA	410.01 ± 2.29aA	214.29 ± 3.61aA	516.88 ± 5.56aA	329.69 ± 9.99aA	19.39 ± 3.93aA	12.84 ± 0.23aA	28.80 ± 0.19aA
	CC	N0	2.27 ± 0.04cB	1.25 ± 0.04bB	278.32 ± 0.65bA	128.93 ± 5.12cB	361.64 ± 5.40cB	256.89 ± 5.16cA	22.07 ± 0.10aA	5.02 ± 0.02bA	16.08 ± 0.38cB
		N180	2.53 ± 0.03bB	1.41 ± 0.02aA	288.48 ± 5.45bA	167.96 ± 6.20bA	375.56 ± 1.26bB	289.62 ± 12.76bA	16.81 ± 4.49aA	7.11 ± 0.02bA	23.29 ± 0.21bB
		N360	2.72 ± 0.05aB	1.48 ± 0.05aB	318.05 ± 4.94aB	209.27 ± 2.95aB	389.64 ± 0.99aB	326.01 ± 1.09aB	20.67 ± 6.01aA	9.88 ± 0.05aB	27.45 ± 0.25aB
15-30cm	RC	N0	2.33 ± 0.12cA	1.30 ± 0.12bA	236.59 ± 0.38cA	127.03 ± 2.98bA	423.57 ± 2.11bA	199.33 ± 1.50bA	14.55 ± 1.59aA	4.92 ± 0.01cA	22.71 ± 0.11bA
		N180	2.63 ± 0.03bA	1.50 ± 0.03abA	340.98 ± 2.65bA	157.69 ± 1.41aA	427.07 ± 1.25bA	245.51 ± 18.31aA	24.83 ± 6.40aA	8.85 ± 0.13bA	22.97 ± 0.07bA
		N360	2.99 ± 0.02aA	1.74 ± 0.02aA	372.80 ± 3.20aA	156.98 ± 3.49aA	440.94 ± 3.97aA	278.35 ± 3.71aA	18.31 ± 3.40aA	12.1 ± 0.01aA	27.63 ± 0.04aA
	CC	N0	1.94 ± 0.08cB	1.06 ± 0.08cB	231.09 ± 5.22bB	118.85 ± 1.59bA	348.77 ± 4.76bB	178.83 ± 21.57aA	16.29 ± 1.98aA	4.63 ± 0.12cA	15.62 ± 0.01cB
		N180	2.35 ± 0.02bB	1.34 ± 0.02bB	257.64 ± 1.71aB	174.51 ± 8.33aA	362.36 ± 5.73bB	215.47 ± 8.92aA	10.93 ± 6.88aA	6.59 ± 0.02bB	22.92 ± 0.13bA
		N360	2.68 ± 0.04aB	1.62 ± 0.05aA	266.04 ± 5.97aB	191.79 ± 4.92aB	380.72 ± 2.63aB	219.17 ± 7.05aB	19.26 ± 4.26aA	10.14 ± 0.49aB	27.57 ± 1.24aA

Lowercase letters represent significant differences (p<0.05) between different N application rates for the same treatment in the same year; uppercase letters represent significant differences (p<0.05) in corn yield between RC and CC under the same N application rate in the same year. TN, total nitrogen; NHN, non-acid hydrolyzed nitrogen; AAN, aminonium acid nitrogen; ASN, aminonium sugar nitrogen; AN, acid-hydrolyzable ammonium nitrogen; UN, acid hydrolyzable unknown nitrogen; MBN, microbial biomass nitrogen; NH_4_^+^-N, ammonium nitrogen; NO_3_^−^-N, nitrate nitrogen.

Soil mineral N is the main form of N taken up by crops. The nitrate N content was significantly higher than the ammoniacal N content, which increased with increasing N application rate. In the 0-15 cm soil layer and at the N360 level, the ammoniacal N content in the RC treatment was 2.96 mg kg^-1^ higher than that in the CC treatment, and the difference was significant. The differences between the other treatments were not significant. The nitrate N content in the RC treatment was significantly higher than that in the CC treatment, and the increase in nitrate N was in the range of 0.92-6.79 mg kg^-1^. In the N180 and N360 treatments and in the 15-30 cm soil layer, the ammoniacal N content in the RC treatment was significantly greater than that in the CC treatment, with increases of 2.26 mg kg-1 and 1.96 mg kg^-1^, respectively. At the N0 level, the nitrate N content in the RC treatment increased by 5.09 mg kg^-1^ compared to the CC treatment, and the difference was significant.

The activities of soil N-transforming enzymes increased gradually increased with N fertilizer (**Table 5**). At the same N fertilization level and in the same soil layer, the soil enzyme activities in the RC treatment were significantly higher than those in the CC treatment, indicating that preceding soybean cultivation significantly increased the activities of enzymes related to soil N transformation and improved the N supply capacity in the soil. Moreover, the enzyme activity in the 0-15 cm soil layer was greater than that in the 15-30 cm soil layer, indicating that the N turnover efficiency of the surface soil was greater. The enzyme activities of NAG, urease, NR, NiR, and LAP in the RC treatment increased by 0.005-2.72U g^-1^, 38.58-147.94U g^-1^, 0.28-0.54U g^-1^, 5.89-18.49U g^-1^ and 0.19-0.47U g^-1^, respectively, in the 0-15 cm soil layer, and increased by 0.13-1.63U g^-1^, 44.17-119.53U g^-1^, 0.38-0.48U g^-1^, 7.55-10.47U g^-1^ and 0.10-0.37U g^-1^, respectively, in the 15 cm-30 cm layer, compared to those in the CC treatment.

**Table 5:** Enzymes related to soil N transformation (U g^-1^).

Soil depth	Treatments	N level	NAG	Urease	NR	NiR	LAP
0-15cm	RC	N0	4.07 ± 0.08cA	508.63 ± 6.63cA	1.21 ± 0.05bA	49.37 ± 1.78bA	1.41 ± 0.06cA
		N180	4.82 ± 0.04bA	683.33 ± 9.76bA	1.63 ± 0.21bA	53.84 ± 1.14bA	1.67 ± 0.04bA
		N360	9.01 ± 0.36aA	929.72 ± 4.26aA	2.40 ± 0.09aA	65.02 ± 1.77aA	2.05 ± 0.03aA
	CC	N0	4.02 ± 0.05cA	470.05 ± 9.50cB	0.86 ± 0.04cB	43.48 ± 1.87aA	1.22 ± 0.02cB
		N180	4.70 ± 0.04bA	543.92 ± 21.60bB	1.35 ± 0.03bA	46.20 ± 1.16aB	1.44 ± 0.05bB
		N360	6.29 ± 0.17aB	781.78 ± 25.31aB	1.86 ± 0.14aB	46.53 ± 1.27aB	1.58 ± 0.01aB
15-30cm	RC	N0	3.97 ± 0.26bA	428.61 ± 25.94cA	1.15 ± 0.1cA	49.55 ± 1.53bA	1.23 ± 0.05cA
		N180	4.52 ± 0.12bA	579.73 ± 18.74bA	1.53 ± 0.06bA	53.30 ± 2.14bA	1.53 ± 0.03bA
		N360	7.11 ± 0.16aA	848.28 ± 25.89aA	2.18 ± 0.11aA	59.17 ± 0.55aA	1.91 ± 0.03aA
	CC	N0	2.67 ± 0.06cB	384.44 ± 18.01cA	0.77 ± 0.03cB	42.00 ± 1.33bB	1.13 ± 0.01cA
		N180	4.39 ± 0.08bA	520.17 ± 13.11bB	1.32 ± 0.03bA	43.75 ± 0.71bB	1.43 ± 0.03bA
		N360	5.48 ± 0.12aB	729.75 ± 26.51aB	1.70 ± 0.05aB	48.70 ± 1.15aB	1.54 ± 0.03aB

Lowercase letters represent significant differences (p<0.05) between different N application rates for the same treatment in the same year; uppercase letters represent significant differences (p<0.05) in corn yield between RC and CC under the same N application rate in the same year.

**Figure 6:**
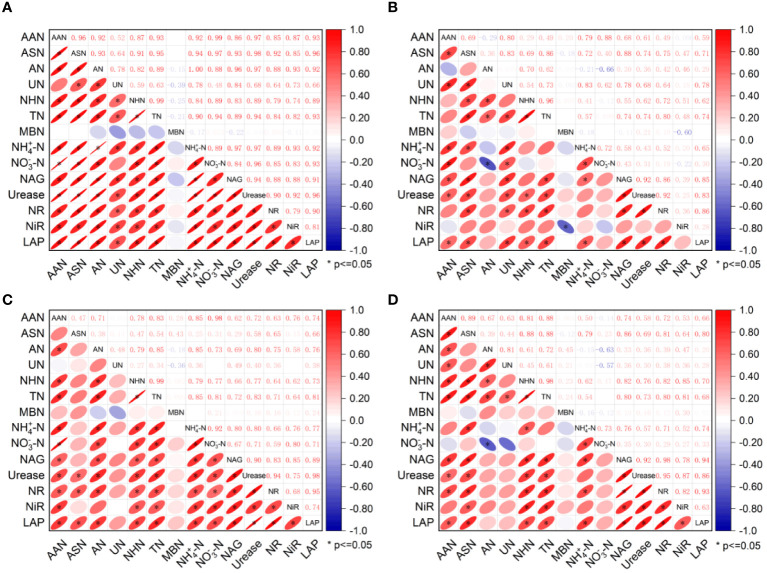
Plant N accumulation and NUE. **(A)** shows the 0-15 cm soil layer in the RC treatment, **(B)** shows the 0-15 cm soil layer in the CC treatment, **(C)** shows the 15-30 cm soil layer in the RC treatment, and **(D)** shows the 15-30 cm soil layer in the CC treatment.

An analysis of the N content and N transformation related enzyme activities in the two soil layers and two crops showed that with the exception of MBN, most soil N components and enzymes were significantly correlated (**Figure 6**). In particular, in the 0-15 cm soil layer of the RC treatment, a significant positive correlation was detected between the N components and enzyme activities (**Figure 6A**). In the 0-15 cm soil layer of the CC treatment, four indicators, AN, NO_3_^−^-N, NH_4_^+^-N, and NiR, correlated poorly with the other components (**Figure 6B**). In the 15-30 cm soil layer of the RC treatment, except for ASN, UN, and MBN, the correlations between the other indicators were greater (**Figure 6C**). In the 15-30 cm soil layer of the CC treatment, AN, UN, MBN, and NO_3_^−^-N were poorly correlated with the other indicators (**Figure 6D**).

### 3.4 Yield increase effect and soil N stock

**Figure 7:**
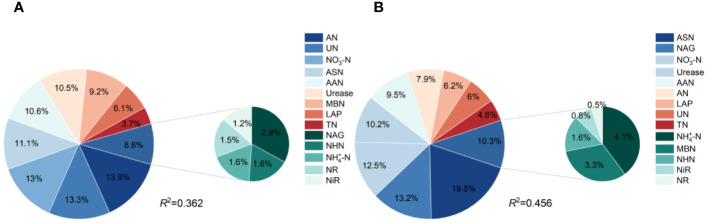
Contribution rates of the N components to yield. **(A)** is the contribution rate of changes in soil N indicators in the 0-15 cm soil layer to yield increase; **(B)** is the contribution rate of changes in soil N indicators in the 15-30 cm soil layer to yield increase.

Based on the differences in the soil N indicators and yield differences between the RC and CC treatments, a random forest model was used to analyze the contributions of changes in the different indicators to yield changes (**Figure 7**). Specifically, the soil indicators in the 0-15 cm soil layer whose changes contributed more than 10% to the yield were AN, UN, NO_3_^−^-N, ASN, AAN and urease (**Figure 7A**), while in the 15-30 cm soil layer, they were ASN, NAG, NO_3_^−^-N, and urease (**Figure 7B**). In the two soil layers, NO_3_^−^-N, ASN and urease were important indicators of corn yield increase, and the average contribution rates to corn yield increase were 12.75%, 15.30% and 10.35%, respectively.

**Figure 8:**
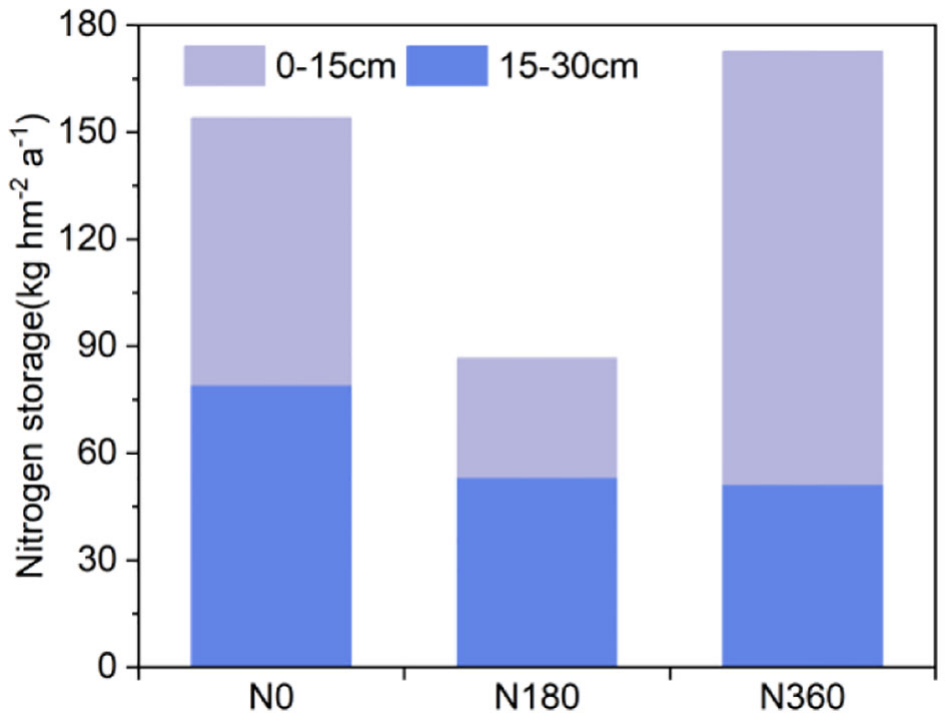
Estimation of N stocks.

Based on the soil TN content (**Table 4**) and soil bulk density (**Supplementary Table 1**), the increase in N fixation per unit area under the same N level between the RC and CC treatments was estimated. In this study, the RC treatment was applied for a total of 3 years, with the rotation cycle starting with soybean cultivation in 2017, the increase in soil N stock per unit area in each rotation cycle (2 years) was calculated by the Equations (1) and (2). In each rotation cycle (2 years), the N fixation per unit area of N0, N180 and N360 in the RC treatment increased by 149.7 kg hm^-2^, 66.75 kg hm^-2^ and 242.7 kg hm^-2^, respectively, in the 0-15 cm soil layer, and by 158.3 kg hm^-2^, 106.45 kg hm^-2^ and 102.5 kg hm^-2^, respectively, in the 15 cm-30 cm layer, compared to that in the CC treatment. The N fixation per unit area in the 0-30 cm tillage layer of corn and soybeans planted alternately was 86.6-172.6 kg hm^-2^. Excessive N fertilizer application in corn-soybean rotation significantly increased N stock in the 0-15 cm soil layer (**Figure 8**).

## 4 Discussion

### 4.1 The impact of crop rotation systems on yield and soil nitrogen content

Crop rotation is considered an environmentally friendly strategy for sustainable agriculture. It effectively manages nutrients, water, weeds, and pests, while maintaining soil structure and fertility, thereby enhancing crop yields (Tilman et al., 2002; Castellazzi et al., 2008; Bender et al., 2016; German et al., 2017). In particular, cereal-legume rotations (nutrient-rich preceding crops) are the most favored, significantly boosting the yield of subsequent crops by up to 27%. This is well-supported by previous studies involving quantitative meta-analyses based on local field experiment data (Preissel et al., 2015; Cernay et al., 2018; Franke et al., 2018). In this study, under two nitrogen application levels (N180 and N360), the yield increased from planting soybeans as the previous crop compared to continuous maize cropping ranged from 4.76% to 39.03%, further confirming the yield-enhancing effect of soybean as a precursor crop for maize. This further confirms the yield-boosting effect of soybean as a preceding crop for maize. Research by Evans et al. (1991) and Zang et al. (2015) has demonstrated that legume-based rotation systems yield 14% more than systems without legumes, and it is mainly due to the nitrogen-fixing ability of legumes and the nitrogen-rich residues they leave behind, which significantly enhance nitrogen input for subsequent crops. Moreover, in this study, the impact of preceding crops on the yield and nutrient content of subsequent crops varied significantly. Soybean as a preceding crop notably increased the dry matter weight and nitrogen content of maize plants. This rotation system significantly reduced the stover-to-grain ratio in maize, leading to differences in dry matter distribution among grains, stems, and leaves.

The maize-soybean rotation improves the biochemical properties of the soil. Biological nitrogen fixation (BNF) is a significant way through which soybean plants increase soil nitrogen content (Thorup-Kristensen et al., 2003) and is crucial for stabilizing soil nutrient supply (Jantalia et al., 2007). In this study, the maize-soybean rotation significantly increased total nitrogen (TN) content in the soil, ensuring an adequate supply of available nitrogen. In the 0-15 cm and 15-30 cm soil layers, the TN content in the rotation treatment (RC) was significantly higher than in the continuous cropping treatment (CC). Additionally, the RC treatment reduced the proportion of acidolysis unknown nitrogen (UN) and the difficulty of soil organic nitrogen mineralization, while increasing the proportion of amminia nitrogen (**Supplementary Figure 2**). This is important because crop nitrogen uptake primarily relies on mineral nitrogen, which is a key indicator of soil nitrogen supply capacity. Soybeans in the rotation system exhibit high recovery capabilities for NH_4_^+^ and NO_3_^-^ (Zhang et al., 2008; Shen et al., 2018). In this study, the content of ammonium nitrogen and nitrate nitrogen in the 0-15 cm soil layer was significantly higher in the maize-soybean rotation treatment compared to continuous maize cropping. This increase provided more soil-based mineral nitrogen for the subsequent maize crop, reducing the need for nitrogen fertilizer input. Peoples et al. (2009) and Barcelos et al. (2022) also found that legumes could fix approximately 39-182 kg N ha^−1^, and the increase in soil nitrogen reserves could regulate nitrogen fertilizer application. When fertilizer input is low during production, the rotation treatment demonstrates a stronger nutrient supply capacity due to the high nutrient demand of cereals. This aligns with our findings that maize yields were relatively high even when no nitrogen fertilizer was applied to the preceding soybean crop (**Figure 3**) (Xu et al., 2020; Mahmoud et al., 2022).

### 4.2 The impact of nitrogen application rates on the content of soil organic nitrogen components

The content of soil organic nitrogen (SON) is of great significance for maintaining soil nitrogen fertility and determining soil nitrogen supply capacity. It is an important indicator of soil fertility (Tang et al., 2021). The composition, content, and ease of mineralization of soil organic nitrogen are closely related to soil nitrogen supply characteristics (Wu et al., 2021). Numerous studies have shown that the amount of nitrogen applied is a significant factor affecting the content of soil organic nitrogen components (He et al., 2018; Li et al., 2019; Sebastian et al., 2023). This finding aligns with our study, which also demonstrates that different levels of nitrogen fertilizer application in both rotation and continuous cropping systems significantly alter the composition of soil organic nitrogen components (**Table 4**). However, the relationship between nitrogen application rates and various soil organic nitrogen components is not entirely consistent with literature reports. Many studies suggested that acid-hydrolyzable non-protein nitrogen (ANN) and acid-hydrolyzable ammonium nitrogen (AN) were readily consumed during soil total nitrogen depletion (He et al., 2018; Ma et al., 2020; Tang et al., 2021), while other studies reported no impact of fertilization on ANN and AN content. For instance, Gao et al. (2009) found no significant effect of chemical fertilizers on the content of organic nitrogen and its components in a 29-year maize-maize-soybean rotation system in Shenyang. Similarly, Zhang et al. (2015) reported no significant effect of nitrogen application rates (180, 225, 270 kg/hm²) on soil total nitrogen and organic nitrogen components in a 7-year wheat-maize rotation system in Hebi, Henan.

In contrast, this study found that nitrogen application rates directly affect the content of ANN and amino sugar nitrogen (ASN) in the maize-soybean rotation system. This phenomenon may be attributed to factors such as the saturation level of adsorption sites on soil particles for organic nitrogen components and the nitrogen fertilizer application level. ANN primarily originates from soil fixed ammonium, while ASN mainly derives from the degradation of soil microbial cell walls. The content of these components is related to the retention capacity of soil colloids. In this study, long-term high fertilization in arable land accumulated high levels of soil organic nitrogen components. The stronger interactions among different components in competition for adsorption sites might be influenced by the adsorption retention capacity of soil colloids, playing a key role in the changes in component content.

ASN is a major component of total acid-hydrolyzable nitrogen, and its content responds significantly to changes in nitrogen application rates. Experimental results indicated that ASN content in the soil could reach 157.69-214.29 mg/kg. This finding is not entirely consistent with some literature reports. For example, Tang et al. (2021) and Jiao et al. (2020) reported that ASN accounted for a relatively low proportion of TNex (total nitrogen extractable) and showed a low response to changes in nitrogen application rates. However, other studies suggested that ASN could have higher concentrations in the soil. Hao et al. (2015) reported that ASN content could reach 175.1 mg/kg when organic and inorganic fertilizers were used in combination on black soil in Northeast China. Similarly, Ji et al. (2020) found that ASN content could reach 152.62 mg/kg in black soil surveys. Wu et al. (2021) also reported that ASN could reach 137.95 mg/kg in no-tillage maize fields in Northeast China. These results indicate that ASN can accumulate in high-yield, high-input systems. High crop yields result in more organic matter residues in the soil and higher microbial activity, leading to the accumulation of ASN derived from microbial residues in the soil (Zhao et al., 2014; Ma et al., 2018).

### 4.3 The impact of the maize-soybean rotation system on the content of soil organic nitrogen components

Crop rotation can improve soil conditions, promote the proliferation of beneficial microorganisms, and increase nitrogen mineralization and nitrification processes, thereby enhancing the availability of nitrogen in the soil (Yuan et al., 2022). This study found that the content of soil nitrogen components was significantly higher under the soybean rotation treatment compared to continuous maize cropping, consistent with the findings of Yuan et al. (2022). This is because crop rotation helps to promote soil microbial diversity and activity. Nitrogen forms such as acid hydrolyzable ammonium nitrogen and acid hydrolyzable unknown nitrogen contribute to the structural and functional composition of plants, support microbial activity in the soil, and enhance soil fertility and health (Li et al., 2021).

Crop rotation significantly increased soil nitrogen reserves (**Supplementary Table 2**), although the annual average increased in soil nitrogen reserves varied significantly under different nitrogen levels. Notably, under the N0 treatment, the nitrogen reserve in the 0-30 cm soil layer was 67.4 kg/ha higher than that under the N180 treatment. This finding aligns with the results of Lin et al. (2021) and Salvagiotti et al. (2008) which indicate that low nitrogen input conditions enhance the nitrogen-fixing effect of preceding soybean crops. The main difference in nitrogen reserves between the N360 and N180 levels was observed in the 0-15 cm soil layer. We believe that the continuous high nitrogen fertilizer application is the primary cause of this difference. Returning crop residues to the field further adjusted the soil carbon-to-nitrogen ratio, triggering the mineralization of natural organic carbon in the short term and enhancing the soil’s nitrogen fixation capacity (Guenet et al., 2010; Kirkby et al., 2014; Li et al., 2022).

This study measured the microbiological characteristics of the 0-30 cm soil layer and found that urease and microbial biomass nitrogen (MBN) significantly increased under the rotation treatment. This might be due to the soluble carbon (C) and nitrogen (N) released from crop residues, which enhanced microbial activity, thereby improving nitrogen mineralization and supply capacity (Yang et al., 2021). We observed that in the maize-soybean rotation system, the activity of nitrogen transformation-related enzymes increased correspondingly with higher nitrogen fertilizer application rates. Similarly. The experiments with different tillage methods and crop residue incorporation of Yang (2023) showed significant enhancement in nitrogen transformation-related enzyme activity in the wheat-peanut rotation system. These findings further reflect the positive impact of crop rotation and nitrogen fertilizer application on soil enzyme activity. The practice of crop rotation combined with crop residue incorporation can alter the source of soil nutrients, leading to enhanced microbial communities and soil enzyme activity (Yang et al., 2021). The growth of certain microbial groups in the soil, such as oligotrophic bacteria, may help utilize nutrients from soil organic matter (SOM) and, following crop rotation and residue incorporation, promote microbial utilization of soil nutrients (Fierer et al., 2007; McDaniel and Grandy, 2016). In this study, enzyme activity in the RC treatment was greater than in the CC treatment, indicating that residues from preceding soybean crops favor microbial proliferation and the production of more enzymes (Lian et al., 2019). The increase in soil microbial and enzyme activity can promote the transformation of crop residues into soil organic matter (SOM), enhance nutrient use efficiency, and partially replace nitrogen fertilizers. This improvement is reflected in soil nutrient content and maize yield (Wei et al., 2019).

### 4.4 The effect of soybean precursors on improving nitrogen fertilizer use efficiency

The nitrogen fertilizer applied to the soil has three possible fates: being absorbed by the crop, remaining in the soil, and being lost from the system (Ruisi et al., 2016). This study found that the dry matter weight of maize plants increased with the amount of fertilizer applied, and the abundance of ^15^N showed a similar trend. The highest ^15^N abundance was observed at the seedling stage of maize, gradually decreasing as the plants grew. This indicates that maize plants rely more on fertilizer nitrogen during the seedling stage (Jiang et al., 2011). After the tasseling stage, plants absorbed more soil nitrogen, as evidenced by the rapid accumulation of plant dry matter and the gradual decrease in ^15^N abundance.

At the seedling stage, the impact of nitrogen fertilization on root ^15^N abundance was relatively small, while the ^15^N abundance in the stems and leaves of the CC treatment was significantly higher than that of the RC treatment. This indicates that continuous maize cropping requires more fertilizer nitrogen (Gentry et al., 2013). The maize-soybean rotation altered the form and proportion of nitrogen in the soil and increased the soil’s nitrogen supply capacity (Li et al., 2013), as reflected by higher stem and leaf nitrogen content and lower ^15^N abundance in the RC treatment. Notably, in the RC treatment, the ^15^N abundance in the roots at the seedling stage was significantly higher than in the stems and leaves (**Table 2**). Combined with soil nitrogen composition data, this study suggests that the soil nitrogen supply in the preceding soybean crop treatment is more conducive to promote plant growth and increase yield (Armstrong et al., 1997).

Nitrogen fertilizer plays a crucial role in promoting soybean and maize growth and increasing grain yield (Jing et al., 2009). However, in the black soil regions of Northeast China, nitrogen fertilizer application exceeding 250 kg ha^-1^ for maize leds to decrease nitrogen use efficiency (NUE) as nitrogen application increased (Li et al., 2022), especially with additional basal nitrogen fertilization aftered maize straw return, which could significantly reduce NUE (Tian et al., 2018). Therefore, in high-input agricultural areas, it is recommended to control nitrogen fertilizer application to improve fertilizer efficiency. However, reducing nitrogen fertilizer application while maintaining high yields remains a global challenge.

In suitable ecological zones, crop rotation involving legumes like soybeans is an effective strategy to improve fertilizer efficiency (Ju et al., 2009). In Northeast China, excessive pursuit of crop yields by producers has led to severe overuse of fertilizers in the past 20 years (Geng et al., 2023). In this study, planting maize after preceding soybeans at the N180 level achieved both high yield and high nitrogen use efficiency. Importantly, nitrogen fertilizer use efficiency in continuous maize cropping was significantly lower compared to the preceding soybean treatment. Similar findings by Wu et al., 2022 suggested that for maintaining high yields and improving NUE, rational nitrogen fertilization under crop rotation with straw return was advisable.

## 5 Conclusions

The preceding soybean crop significantly increased the yield of the subsequent corn crop, and the yield increase was significantly influenced by the amount of N applied. Both preceding soybean plants and N fertilization increased the activities of soil N-transforming enzymes, which in turn increased the soil N content, improved the proportions of soil organic N components, and increased the soil N stocks; allowing N fertilizer inputs for the succeeding crop to be reduced. A corn-soybean rotation with low N input is an effective and sustainable strategy for increasing soil NUE, improving soil N supply characteristics and maintaining the stability of agricultural production.

## Data availability statement

The original contributions presented in the study are included in the article/**Supplementary Material**. Further inquiries can be directed to the corresponding author.

## Author contributions

CY: Writing – review & editing, Data curation, Formal analysis, Funding acquisition, Methodology, Visualization, Writing – original draft. YY: Data curation, Writing – original draft, Investigation, Validation. JS: Data curation, Investigation, Validation, Writing – original draft. FS: Data curation, Validation, Writing – original draft, Visualization. XL: Data curation, Validation, Visualization, Writing – review & editing. SY: Validation, Visualization, Writing – review & editing. CW: Validation, Visualization, Writing – review & editing. QS: Writing – review & editing, Conceptualization, Project administration, Resources. CM: Writing – review & editing, Conceptualization, Project administration, Resources.
