# Supplementary material for: Analysis of the beneficial effects of prior soybean cultivation to the field on corn yield and soil nitrogen content
Source: Front Plant Sci. 2024 Jul 30;15:1413507. doi: 10.3389/fpls.2024.1413507 (PMC11319277; doi:10.3389/fpls.2024.1413507)
Supplement: Supplementary file 1 [file Table_1.docx]

Supplementary Material

Analysis of the beneficial effects of prior soybean cultivation to the field on corn yield and soil nitrogen content

Chao Yan^1^, Yi Yang^1^, Junming Song^2^, Fuxin Shan^1^, Xiaochen Lyu^1^, Shuangshuang Yan^1^, Chang Wang^1^, Chunmei Ma^1^*

^1^College of Agriculture, Northeast Agricultural University, Harbin 150030, China

^2^Chinese People’s Armed Police Force Non Commissioned Officer School, Hangzhou, 310000, China

*** Correspondence:**Chunmei Ma
chunmm@neau.edu.cn

**Supplementary Table 1.** Soil bulk density（g cm^-3^）

| Soil depth | N level | RC | CC |
| --- | --- | --- | --- |
| 0-15 cm | N0 | 1.05±0.02 | 1.08±0.01 |
|  | N180 | 1.04±0.01 | 1.09±0.01 |
|  | N360 | 1.05±0.01 | 1.08±0.02 |
| 15-30 cm | N0 | 1.16±0.01 | 1.23±0.01 |
|  | N180 | 1.18±0.02 | 1.23±0.02 |
|  | N360 | 1.18±0.01 | 1.24±0.01 |

**Supplementary Table 2.** Soil nitrogen stocks（kg hm^-2^）

| Soil depth | N level | RC | CC |
| --- | --- | --- | --- |
| 0-15 | N0 | 4126.5±28.98cA | 3677.4±35.48cB |
|  | N180 | 4336.8±21.34bA | 4136.55±26.13bB |
|  | N360 | 5134.5±22.9aA | 4406.4±28.04aB |
| 15-30 | N0 | 4054.2±38.41cA | 3579.3±23.52cB |
|  | N180 | 4655.1±46.6bA | 4335.75±28.53bB |
|  | N360 | 5292.3±34.39aA | 4984.8±21.05aB |

Note: The lowercase letters are the same soil layer treatment with different nitrogen levels p < 0.05 difference significant comparison ; uppercase letters for the same year under the same nitrogen level between RC and CC p < 0.05 significant difference comparison.

**Supplementary Figure 1.** Maize yield components. The lowercase letters are the same year with the same treatment of different nitrogen levels p < 0.05 significant difference comparison ; uppercase letters for the same year under the same nitrogen level of RC and CC maize yield p < 0.05 significant difference comparison ; ns is not significant. * and * * are significant at 0.05 and 0.01 levels, respectively.

**Supplementary Figure 2. Soil organic nitrogen fractions.**
