# Supplementary figures and images for: Analysis of the beneficial effects of prior soybean cultivation to the field on corn yield and soil nitrogen content
Source: Front Plant Sci. 2024 Jul 30;15:1413507. doi: 10.3389/fpls.2024.1413507 (PMC11319277; doi:10.3389/fpls.2024.1413507)

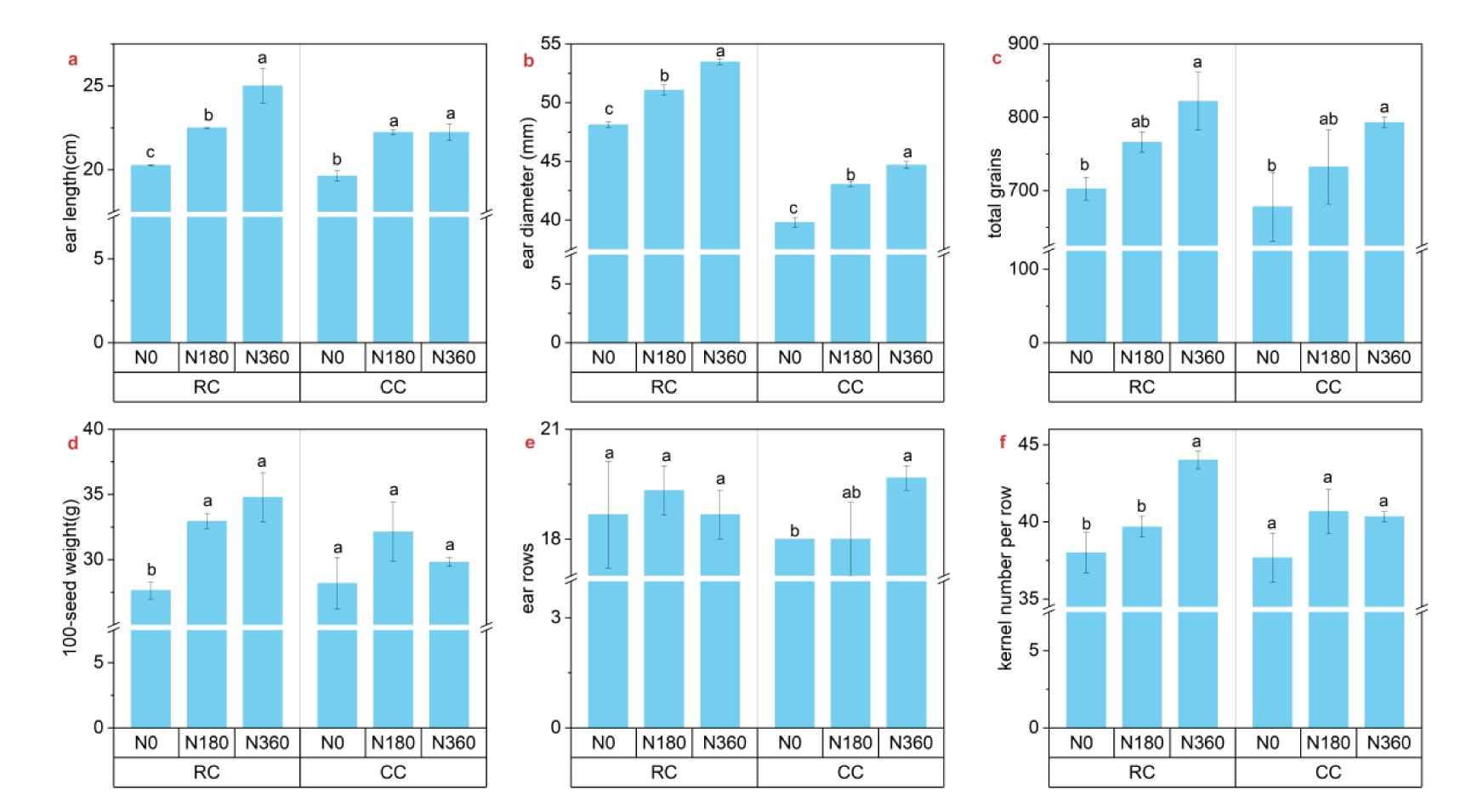

Supplement: Supplementary file 2 [file Image_1.tif]

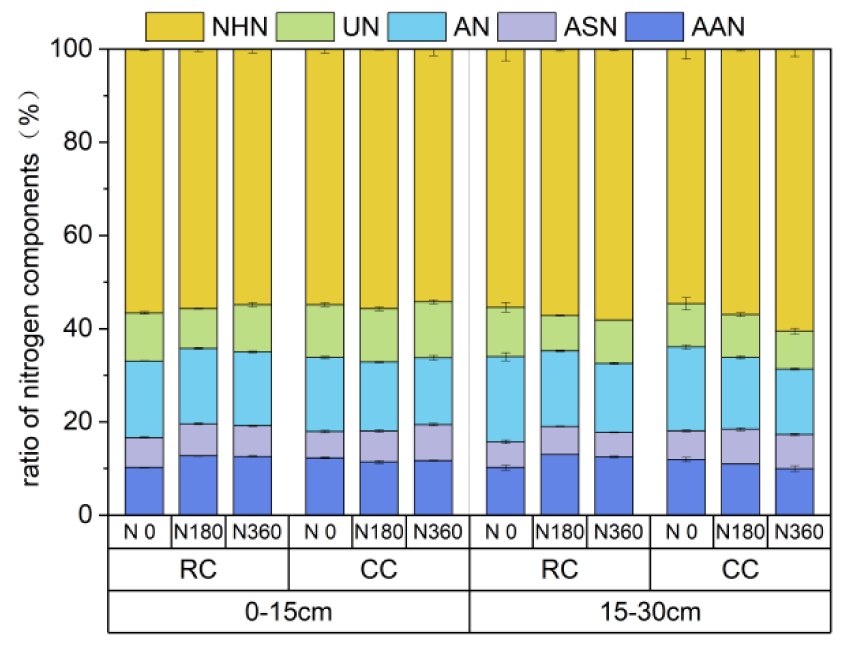

Supplement: Supplementary file 3 [file Image_2.tif]
